# Associations of Dietary Sodium, Potassium, and Sodium to Potassium Ratio with Blood Pressure—Regional Disparities in China

**DOI:** 10.3390/nu12020366

**Published:** 2020-01-30

**Authors:** Lina Huang, Huijun Wang, Zhihong Wang, Yun Wang, Bing Zhang, Gangqiang Ding

**Affiliations:** National Institute for Nutrition and Health, Chinese Center for Disease Control and Prevention, Beijing 100050, China; huangln2999@126.com (L.H.); wanghj@ninh.chinacdc.cn (H.W.); wangzh@ninh.chinacdc.cn (Z.W.); wangyunn@aliyun.com (Y.W.); zhangbing@chinacdc.cn (B.Z.)

**Keywords:** regional disparity, sodium, potassium, blood pressure

## Abstract

High dietary sodium and low potassium intake increase blood pressure and risk of hypertension, but whether the relationship between dietary sodium and potassium and risk of hypertension is different in North China and South China remains unclear. We used data from the longitudinal China Health and Nutrition Survey (CHNS) and selected 6705 adults who participated in at least two waves in 2009, 2011, and 2015 and had no hypertension in baseline. We performed multiple linear regression analysis and multiple logistic regressions stratified by area for the present study design. Sodium and potassium intake were higher in North China (4343.4 and 1624.8 mg/day, respectively) than in South China (4107.8 and 1516.1 mg/d, respectively) (*p* < 0.05). Multiple linear regression revealed that a positive correlation of sodium intake (β = 0.026, *p* < 0.05) and ratio of sodium to potassium (Na-K) intake (β = 0.041, *p* < 0.01) with diastolic blood pressure (DBP) was found in North China, and the association of sodium, potassium, and Na-K intake ratio with blood pressure was different in South China. Multiple logistic regressions documented a similar significant inverse association between dietary potassium intake and risk of hypertension in both North China and South China (risk ratio (RR): 0.63, 95%CI: 0.50–0.79; RR: 0.80, 95%CI: 0.66–0.98, respectively). The risk of hypertension increased in the fourth quartile of dietary sodium and Na-K intake ratio (RR: 1.20, 95%CI: 1.00–1.44; RR: 1.35, 95%CI: 1.13–1.62, respectively) in North China but no association was observed in South China. The current study indicates a different association of dietary sodium and Na-K intake ratio with systolic blood pressure (SBP), DBP, and risk of hypertension in North China and South China.

## 1. Introduction

Hypertension is the leading preventable risk factor for premature death and disability worldwide [1]. In 2010, 31.1% of the global adult population, or about 1.34 billion to 1.44 billion, had hypertension [2]. Overall, 23.2% (estimated 244.5 million) of Chinese adults had hypertension [3]. In 2017, it was the number one risk factor contributing to deaths and disability adjusted life years (DALYs) in China, accounting for 2.54 million deaths of which 95.7% were due to cardiovascular diseases [4]. The World Health Organization (WHO) and other organizations have emphasized the relationship of dietary sodium, potassium, and sodium to potassium ratio with hypertension and cardiovascular disease [5,6,7]. Based on the findings from the China National Health and Nutrition Surveillance (2010–2012), dietary sodium intake was 5702.7 mg/day (per standard person day) among Chinese adults and much higher than the intake recommended by the WHO. Meanwhile, dietary potassium intake (1616.9 mg (per standard person day)) was much lower [8].

Dietary intake varies considerably between populations. There were geographic variations in dietary habits, cooking, social environment, and local foods in South China and North China. Thus, dietary sodium and potassium intake varies by region and this might have a different impact on the risk of developing hypertension. However, few studies have compared the regional disparities in dietary sodium, potassium, and Na-K intake ratio and the risk of hypertension in North China and South China, especially longitudinal studies. In the present study, we used data from CHNS to examine regional disparities in the association between dietary sodium, potassium, and Na-K intake ratio and the risk of hypertension.

## 2. Materials and Methods

### 2.1. Study Design and Subjects

All data used in this study are from the CHNS, an ongoing, large-scale, longitudinal, household-based survey of eleven waves. Before 2009, the survey covered nine provinces (including Jiangsu, Hubei, Hunan, Guangxi, Guizhou, Heilongjiang, Liaoning, Shandong, and Henan). In 2011, the CHNS included the additional provinces of Beijing, Shanghai, and Chongqing, and it included Shanxi, Yunnan, and Zhejiang in 2015. The CHNS and the survey procedure has been described in detail elsewhere [9,10]. Based on the natural boundary of a line along the Qinling Mountains and the Huaihe River, China is divided into North China and South China geographic areas [11]. All the analyses in this study were based on the data from 2009, 2011, and 2015. There were 10,051 respondents who were aged 18 or older when they were recruited and participated in at least two waves in 2009, 2011, and 2015. We excluded pregnant and lactating women (*n* = 0); those previously diagnosed with hypertension (*n* = 3125) in baseline; and those with missing information on diet, blood pressure and covariates (*n* = 221). Finally, there were a total of 6705 (2610 from North China, 4095 from South China) participants in the analysis. 

All participants gave written informed consent before participating in the study. This study was approved by the Institutional Review Board of the University of North Carolina at Chapel Hill and the National Institute for Nutrition and Health, Chinese Center for Disease Control and Prevention (2015017).

### 2.2. Dietary Data

Dietary data in the CHNS was collected through face-to-face interview and based on a combination of three consecutive days of 24-h recalls for each individual, along with a household food inventory over these days, which included two weekdays and one weekend day. For the food inventory, all available purchased and stored foods in the household were measured. Changes in household food inventory as well as wastage were used to estimate total household food consumption. We determined the percentage of the oil, salt, and other condiments from the household inventory that each member consumed by the ratio of their energy intake to the energy intake of all members. Detailed information, including the types of food, amounts of food, types of meal, and eating location of all food items consumed, was recorded in the 24-h dietary recalls. Based on the intake of each ingredient consumed during the three days, we used the China Food Composition Table to estimate the 3-day average of total energy and nutrient. 

### 2.3. Measurements and Definition

Anthropometrical measurements were conducted by well-trained health workers and followed a reference protocol recommended by the World Health Organization. The operation of height and weight measurement is described in detail elsewhere [11]. We calculated the body mass index (BMI) using weight in kilograms divided by measured height in meters squared (kg/m^2^). Participants were advised to avoid alcohol, coffee/tea, and exercise for at least 30 min prior to their blood pressure measurement, and they also took a seated position after at least five minutes of rest before this measurement. SBP and DBP used the average of up to 3 readings obtained under standard conditions using a mercury sphygmomanometer during the physical examination. Hypertension was defined as an average SBP ≥ 140 mm Hg or DBP ≥ 90 mm Hg, and/or currently taking medication to lower blood pressure [12].

Trained interviewers used standard questionnaires to collect information on sociodemographic characteristics, smoking, drinking, physical activity, annual family income, and community. We classified the smoking status as current (Yes) and none/never (No). We categorized the drinking alcohol status as drinking (Yes) and none/never drinking (No). Household incomes were calculated according to household size and grouped into low, middle, and high. Based on 12 multidimensional components reflecting the heterogeneity in economic, social, demographic, and infrastructural characteristics at the community level [13], we calculated the urbanization index and classified it into low, middle, and high. Four domains (occupational, household chore, leisure time, and transportation activities) were included in total physical activity (the method of the calculation is described in detail elsewhere) and we categorized the total physical activity into low, middle, and high [14].

### 2.4. Statistical Analysis

We performed all analyses separately for North China and South China based on statistically significant interactions with sodium intake effect on blood pressure and risk of hypertension (*p* < 0.05). Continuous variables with skewed distribution were analyzed by non-parametric statistical hypothesis testing, including the Wilcoxon signed-rank test and the Kruskal–Wallis test. Collinearity diagnostics were performed before multiple linear regression analysis, and we found that total energy intake, dietary fat, and carbohydrate showed severe collinearity. Using professional judgement, we deleted dietary fat and carbohydrate when estimating the association of blood pressure with dietary sodium, potassium, and Na-K intake ratio by multiple linear regression analysis. We grouped the baseline dietary sodium, potassium, and Na-K intake ratio consumption into quartiles by area, and constructed a series of multiple logistic regression models to assess regional disparities in the association of dietary sodium, potassium, and sNa-K intake ratio with the risk of hypertension. *p* < 0.05 was considered significant. All statistical analyses were performed by the SAS 9.4 (SAS Institute, Inc., Cary, NC, USA).

## 3. Results

### 3.1. Baseline Characteristics of the Study Population 

The baseline characteristics of participants across the quartiles of dietary sodium intake by area are summarized in Table 1. Each quartile of the baseline median sodium intake in North China was significantly higher than that in South China (*p* < 0.05). Meanwhile, each quartile of the baseline median potassium intake in North China was also higher than that in South China, as shown in Table S1. Both sodium and potassium consumption were substantively far from the WHO recommendations. Higher quartiles of sodium intake tended to have higher total energy, protein, dietary fat, and carbohydrate intake in both North China and South China.

### 3.2. Association of Dietary Sodium, Potassium, and Sodium-Potassium Intake Ratio with Blood Pressure

The results of linear regression analyses that examined the relationship between sodium, potassium, Na-K intake ratio, and blood pressure are shown in Table 2. After adjusting for covariates, the SBP (β = −0.095, *p* < 0.01) and DBP (β = −0.095, *p* < 0.01) decreased when potassium intake increased; both sodium and Na-K intake ratio increase were determined to be positively correlated with elevated DBP in North China (*p* < 0.05). In addition, only an association of increased potassium intake with decreased DBP (β = −0.039, *p* < 0.05) was found in South China. More interesting was that there was no significant association of dietary sodium, potassium, and Na-K intake ratio with SBP observed in South China (*p* > 0.05). 

### 3.3. Association of Dietary Sodium, Potassium and Sodium-Potassium Ratio with Risk of Hypertension in North China

Compared with the first quartile, the risk of hypertension increased in the fourth quartile of dietary sodium intake (RR: 1.20, 95%CI: 1.00–1.44) in North China. The risk of hypertension was strongly and inversely associated with the level of dietary potassium consumption (RR: 0.63, 95%CI: 0.50–0.79, *p* < 0.001 for trend). Dietary sodium-potassium ratio was strongly positively associated with the risk of hypertension (RR: 1.35, 95%CI: 1.13–1.62, *p* < 0.01 for trend) (Table 3).

### 3.4. Association of Dietary Sodium, Potassium, and Sodium-Potassium Ratio with Risk of Hypertension in South China

After adjustment for potential confounders, the risk of hypertension increased in the third quartile of dietary sodium intake (RR: 1.19, 95%CI: 1.02–1.38), but there was no significant difference in the fourth quartile when compared with the lowest quartile in South China. The risk of hypertension was also inversely associated with dietary potassium consumption (RR: 0.80, 95%CI: 0.66–0.98). Dietary sodium-potassium ratio showed no association with the risk of hypertension in South China (Table 4).

## 4. Discussion

In the present study, we observed dietary sodium and potassium intake disparities in c and North China. There was a similar significant inverse association between dietary potassium intake and SBP, DBP, and hypertension in both South China and North China. More interestingly, there was a different association of dietary sodium and Na-K intake ratio with DBP and hypertension between South China and North China. We found a significant positive association of dietary sodium and Na-K intake with DBP and hypertension in North China, but no significant association was observed in North China.

The evidence relating high dietary sodium intake and low potassium intake to elevated blood pressure and the development of hypertension is compelling [15]. Dietary interventions have demonstrated their ability to reduce blood pressure in humans. Potassium supplementation in hypertensives was generally associated with decreased blood pressure (decreased SBP of 4.48 mm Hg, DBP of 2.96 mm Hg), especially in high sodium consumers [16]. More evidence has demonstrated that an increased potassium intake not only reduces blood pressure in those with hypertension, but also reduces the risk of stroke, cardiovascular disease, and mortality [17,18]. To date, many studies, including meta-analyses and systematic reviews, have shown that dietary sodium and Na-K intake ratio have varying degrees of effect on SBP and DBP [19,20,21]. Our results are consistent with these previous studies. In addition, several potential factors might explain the results of the different associations of dietary sodium and sodium to potassium ratio with SBP, DBP, and hypertension in South China and North China. First, the structures of dietary consumption in north and south China were different [11,22]. Therefore, their contribution to sodium and potassium intake might differ. Second, salt is a primary source of sodium. Salt consumption is influenced by taste, culture, social custom, the widespread availability of salt, and habit, independent of the need for salt. There were geographic variations in dietary habits, cooking, social environment, and local foods in South China and North China. Salt appetite in North China was higher than that in South China. Zhao et al. also documented much higher salt and Na-K ration intake in North China than in South China [23,24]. Regional variations in sodium, potassium and blood pressure are also apparent in other countries. The National Health and Nutritional Examination Survey III revealed that the south of the United States had higher sodium intake and lower potassium intake than other areas in the United States, and blood pressure and prevalence of hypertension were higher than in the north of the United States [25]. The results from the current study are consistent with these previous studies, and showed positive association of sodium and Na-K intake ratio with elevated blood pressure and a development of hypertension in the north, but no significant association in the south. Furthermore, genetic or shared environmental factors, or both, might determine and influence blood pressure responses to dietary sodium and potassium intake. Gu and colleagues documented that blood pressure response to dietary sodium and potassium intake was hereditary in the Chinese population [26,27]. Several genetic features in the adiponectin gene were associated with blood pressure responses to dietary sodium and potassium intake in the Chinese Han population; the adiponectin gene may contribute to the development of salt sensitivity and potassium sensitivity of BP [28]. The population of salt sensitivity of blood pressure (SSBP) in China differed in different areas, with the distribution frequency of SSBP being 30.4% in North China [29]. Consequently, gene-gene and gene-environment interactions are likely to be relevant in blood pressure response to dietary sodium, potassium, and Na-K intake ratio.

There are limitations to the current study. We divided areas according to the province which participants lived in at the time of the survey, rather than their ancestral home, which made it difficult to distinguish northern or southern people, and we could not discover whether southerners living in the north or northerners living in the south change their taste and dietary habits. In addition, the booming food industry has led to excess habitual consumption of sodium derived largely from commercially processed food products. Sodium consumption from pre-packaged foods was included only fractionally in this study, such as packaged noodles, instant noodles, and breads, etc. Thus, sodium consumption might be greatly underestimated. Besides, recall biases and a potential underestimation or overestimation in dietary recalls are expected as these were self-reported measurements by the participants. Moreover, the intermediate indicators of 24-h urine sodium and potassium were not discussed in the present study. Furthermore, the CHNS sample was not representative of China and the sample size of this study is also relatively small, so we had difficulty generalizing the study’s results. Further studies are required to validate our findings in a larger and more diverse sample and to elucidate the mechanisms underlying the geographic disparities in blood pressure responses to dietary sodium and potassium intake.

## 5. Conclusions

In summary, the current study indicates a different association of dietary sodium and Na-K intake ratio with elevated blood pressure and development of hypertension in South China and North China. These possible regional disparities should be considered in hypertension prevention and treatment, dietary interventions, and when making dietary recommendations.

## Figures and Tables

**Table 1:** Baseline characteristics of participants according to areas quartiles of dietary sodium intake.

Characteristics		North China				*p*	South China				*p*
		Q1	Q2	Q3	Q4		Q1	Q2	Q3	Q4	
N		652	653	653	652		1023	1024	1024	1024	
Sodium (mg/d)		2302.9 (1643.6, 2732.7)	3733.1 (3396.9, 4028.5)	5114.6 (4725.9, 5613.7)	8041.5 (6821.2, 10851.1)	<0.0001	2163 (1544.7, 2591.6)	3510.3 (3251.4, 3798.8)	4795.6 (4427.8, 5222.6)	7627.2 (6589.5, 9884.3)	<0.0001
Age (years) ^a^		46.1 ± 13.4	46.6 ± 13.4	46.1 ± 13.1	46.5 ± 12.7		48.8 ± 15.1	49.2 ± 14.4	46.9 ± 13.9	46.6 ± 13.6	
Household income, n (%)						0.002					<0.0001
	Low	182 (28.13)	217 (33.33)	234 (36.11)	233 (35.85)		398 (39.48)	368 (36.18)	311 (30.67)	274 (26.97)	
	Medium	222 (34.31)	219 (33.64)	216 (33.33)	208 (32.00)		313 (31.05)	325 (31.96)	343 (33.83)	372 (36.61)	
	High	243 (37.56)	215 (33.03)	198 (30.56)	209 (32.15)		297 (29.46)	324 (31.86)	360 (35.5)	370 (36.42)	
Urbanicity index, n (%)						0.015					0.272
	Low	209 (32.06)	225 (34.46)	204 (31.24)	227 (34.82)		322 (31.48)	341 (33.3)	356 (34.77)	329 (32.13)	
	Medium	201 (30.83)	199 (30.47)	219 (33.54)	248 (38.04)		368 (35.97)	358 (34.96)	350 (34.18)	305 (29.79)	
	High	242 (37.12)	229 (35.07)	230 (35.22)	177 (27.15)		333 (32.55)	325 (31.74)	318 (31.05)	390 (38.09)	
Physical activity, n (%)						0.534					<0.0001
	Low	223 (34.2)	229 (35.07)	204 (31.24)	212 (32.57)		382 (37.34)	378 (36.95)	302 (29.49)	302 (29.49)	
	Medium	227 (34.82)	192 (29.4)	214 (32.77)	238 (36.56)		327 (31.96)	309 (30.21)	378 (36.91)	350 (34.18)	
	High	202 (30.98)	232 (35.53)	235 (35.99)	201 (30.88)		314 (30.69)	336 (32.84)	344 (33.59)	372 (36.33)	
Smoking, n (%)						<0.0001					<0.0001
	No	517 (79.29)	470 (71.98)	461 (70.6)	412(63.19)		750 (73.31)	737 (71.97)	692 (67.58)	672 (65.63)	
	Yes	135 (20.71)	182 (27.87)	191 (29.25)	240(36.81)		273 (26.69)	287 (28.03)	330 (32.23)	352 (34.38)	
Drinking, n (%)						<0.0001					<0.0001
	No	488 (74.85)	448 (68.61)	425 (65.08)	370 (56.75)		748 (73.12)	725 (70.8)	666 (65.04)	600 (58.59)	
	Yes	164 (25.15)	205 (31.39)	228 (34.92)	282 (43.25)		275 (26.88)	298 (29.1)	357 (34.86)	424 (41.41)	
BMI (kg/m^2^) ^b^		23 (21.1, 25.4)	23.3 (21.3, 25.7)	23.4 (21.3, 25.7)	23.8 (21.5,26)	0.000	22.2 (20.2, 24.2)	22.2 (20.2, 24.6)	22.2 (20.5, 24.6)	22.7 (20.8, 24.9)	0.007
SBP (mmHg) ^b^		119 (110, 122)	119 (110, 122)	120 (111, 125)	120 (110,124)	0.149	116 (108, 122)	118 (109.5, 124)	118 (108, 124)	117 (109, 124)	0.002
DBP (mmHg) ^b^		79 (71, 81)	80 (72, 81)	80 (72, 81)	79 (71,81)	0.003	75 (70, 80)	75(70, 80)	75 (70, 80)	77 (70, 80)	0.100
Total energy (kcal/d) ^b^		1760.1 (1444.6, 2128.6)	1922.1 (1596.3, 2308.1)	2091.6 (1693.9, 2501.9)	2322 (1864.1, 2778.8)	<0.0001	1758.2 (1366.7, 2181.9)	1981.3 (1612.1, 2375.7)	2173.2 (1720, 2648.6)	2347.9 (1919.7, 2895.1)	<0.0001
Protein (g/d) ^b^		56.8 (44.3, 68.7)	58.4 (47.9, 72.2)	64.1 (52.1, 78.1)	70.9 (56.4, 89.1)	<0.0001	54.7 (43.5, 70.9)	60 (47.7, 74.5)	66.7 (53.3, 82.1)	71.3 (55.4, 91.2)	<0.0001
Dietary fat (g/d) ^b^		55.6 (39.9, 74.4)	62.8 (46.2, 84.3)	72.8 (53.7, 92.9)	82.7 (62.0, 111.9)	<0.0001	62.4 (45.5, 84.3)	73.1 (56.8, 96.3)	82.3 (59.8, 107.8)	98.3 (73.3, 128.4)	<0.0001
Carbohydrate (g/d) ^b^		241.7 (188.6, 307.2)	267.4 (211, 330.3)	272.3 (211.9, 349.9)	291.6 (219.3, 384.3)	<0.0001	227.1 (163.9, 296.2)	249 (188.9, 319.1)	263.7 (193.3, 341.9)	269.5 (189.4, 355.7)	<0.0001
Dietary fiber (g/d) ^b^		10.1 (7.9, 13.7)	10.7 (8.1, 13.6)	11.5 (9.0, 15.4)	12.6 (9.6, 16.9)	<0.0001	7.7 (5.7, 10.9)	8.7 (6.6, 12.2)	9.4 (7.1, 13.5)	10.2 (7.2, 14.9)	<0.0001
Potassium (mg/d) ^b^		1488.9 (1195.1, 1913.5)	1532.8 (1239.0, 1909.1)	1652.1 (1343.7, 2069.3)	1833 (1417.8, 2239.9)	<0.0001	1307.9 (1029.1, 1718.5)	1469.9 (1153, 1846.9)	1563.3 (1262.3, 2048.9)	1739.6 (1347.9, 2205.9)	<0.0001

Abbreviations: Q = quartile, SBP = systolic blood pressure, DBP = diastolic blood pressure. Data of categorical variables expressed as number (%); ^a^ Mean ± SE. ^b^ Median (interquartile ranges) for skewed distribution variables.

**Table 2:** Multiple linear regression analysis of blood pressure with dietary sodium, potassium, and Na-K ratio by regions.

Exposure Factor	SBP ^a^		DBP ^b^	
	Standardized Regression Coefficient (*β*)	*p*-Value	Standardized Regression Coefficient (*β*)	*p*-Value
North China				
Dietary sodium intake	−0.006	0.609	0.026	0.031
Dietary potassium intake	−0.095	0.000	−0.095	0.000
Dietary Na-K intake ratio	0.014	0.199	0.041	0.001
South China				
Dietary sodium intake	0.008	0.385	−0.004	0.706
Dietary potassium intake	−0.011	0.492	−0.039	0.026
Dietary Na-K intake ratio	0.012	0.173	0.007	0.462

Abbreviations: SBP = systolic blood pressure, DBP = diastolic blood pressure. ^a^ Adjusted for gender, age, baseline of household income, urbanicity index, physical activity, smoking, drinking, total energy, protein, dietary fiber, and SBP. ^b^ Adjusted for gender, age, baseline of household income, urbanicity index, physical activity, smoking, drinking, total energy, protein, dietary fiber, and DBP.

**Table 3:** Associations between dietary sodium, potassium, and sodium-potassium ratio and risk of hypertension in North China.

Exposure Factor		Q1	Q2	Q3	Q4	*p-Trend*
Sodium intake						
	N	652	653	653	652	
	Median (Q1, Q3)	2302.9 (1643.6, 2732.7)	3733.1 (3396.9, 4028.5)	5114.6 (4725.9, 5613.7)	8041.5 (6821.2, 10851.1)	
	Model 1	Ref	1.32 (1.12, 1.57) **	1.22 (1.03, 1.45) *	1.30 (1.09, 1.54) **	0.0263
	Model 2 ^a^	Ref	1.31 (1.10, 1.56) **	1.21 (1.02, 1.45) *	1.26 (1.05, 1.5) *	0.079
	Model 3	Ref	1.29 (1.08, 1.53) **	1.20 (1.01, 1.43) *	1.20 (1.00, 1.44) *	0.214
Potassium intake						
	N	653	652	653	652	
	Median (Q1, Q3)	1093.5 (944.2, 1198)	1450.5 (1370.6,1532.2)	1824.7 (1721.9, 1937.2)	2384.6 (2178, 2797.3)	
	Model 1	Ref	0.76 (0.64, 0.9) **	0.88 (0.75, 1.05)	0.87 (0.73, 1.03)	0.3661
	Model 2 ^b^	Ref	0.69 (0.58, 0.82) **	0.72 (0.60, 0.87) **	0.63 (0.50, 0.78) **	0.0004
	Model 3	Ref	0.69 (0.58, 0.82) **	0.72 (0.60, 0.87) **	0.63 (0.50, 0.79) **	0.0004
Na-K ratio						
	N	653	653	652	652	
	Median (Q1, Q3)	1.3 (0.9, 1.6)	2.2 (2.0, 2.5)	3.2 (2.9, 3.5)	5.2 (4.4, 7)	
	Model 1	Ref	1.17 (0.98, 1.39)	1.24 (1.04, 1.47) *	1.32 (1.11, 1.57) **	0.0021
	Model 2 ^c^	Ref	1.17 (0.99, 1.40)	1.27 (1.07, 1.51) **	1.40 (1.18, 1.68) **	0.0002
	Model 3	Ref	1.15 (0.96, 1.37)	1.21 (1.02, 1.45) *	1.35 (1.13, 1.62) **	0.0012

Abbreviations: Q = quartile. Model 1: adjusted for gender, age and baseline of household income, urbanicity index, physical activity, smoking, drinking, SBP, and DBP; ^a^ Model 2: based on Model 1, adjusted for total energy, protein, dietary fat, carbohydrate, dietary fiber, and dietary potassium; ^b^ Model 2: based on Model 1, adjusted for total energy, protein, dietary fat, carbohydrate, dietary fiber, and dietary sodium; ^c^ Model 2: based on Model 1, adjusted for total energy, protein, dietary fat, carbohydrate, and dietary fiber; Model 3: based on Model 2, adjusted for BMI.* *p* < 0.05, ** *p* < 0.01.

**Table 4:** Associations between dietary sodium, potassium, and sodium-potassium ratio and risk of hypertension in South China.

Exposure Factor		Q1	Q2	Q3	Q4	*p-Trend*
Sodium intake						
	N	1023	1024	1024	1024	
	Median (Q1, Q3)	2163.0 (1544.7, 2591.6)	3510.3 (3251.4, 3798.8)	4795.6 (4427.8, 5222.6)	7627.2 (6589.5, 9884.3)	
	Model 1	Ref	1.02 (0.88, 1.19)	1.25 (1.08, 1.45) **	1.11 (0.95, 1.29)	0.106
	Model 2 ^a^	Ref	1.00 (0.86, 1.17)	1.21 (1.04, 1.41) *	1.05 (0.89, 1.23)	0.459
	Model 3	Ref	1.00 (0.86, 1.17)	1.19 (1.02, 1.38) *	1.02 (0.87, 1.20)	0.655
Potassium intake						
	N	1023	1024	1024	1024	
	Median (Q1, Q3)	977.3 (826.1, 1077.4)	1346.2 (1265.8, 1436.0)	1706.4 (1606.8, 1830.8)	2346.1 (2118.6, 2786.8)	
	Model 1	Ref	0.92 (0.79, 1.07)	1.08 (0.93, 1.26)	1.00 (0.86, 1.16)	0.668
	Model 2 ^b^	Ref	0.86 (0.74, 1.01)	0.96 (0.82, 1.14)	0.82 (0.67, 1.00) *	0.109
	Model 3	Ref	0.84 (0.72, 0.98) *	0.94 (0.80, 1.11)	0.80 (0.66, 0.98) *	0.089
Na-K ratio						
	N	1028	1018	1024	1025	
	Median (Q1, Q3)	1.3 (1.0, 1.6)	2.2 (2.0, 2.5)	3.2 (2.9, 3.5)	5.3 (4.5,7.1)	
	Model 1	Ref	1.14 (0.98, 1.32)	0.99 (0.85, 1.16)	1.10 (0.95, 1.28)	0.468
	Model 2 ^c^	Ref	1.13 (0.97, 1.31)	0.99 (0.85, 1.16)	1.11 (0.95, 1.30)	0.389
	Model 3	Ref	1.13 (0.97, 1.31)	0.97 (0.83, 1.14)	1.10 (0.95, 1.29)	0.445

Abbreviations: Q = quartile. Model 1: adjusted for gender, age and baseline of household income, urbanicity index, physical activity, smoking, drinking, SBP, and DBP; ^a^ Model 2: based on Model 1, adjusted for total energy, protein, dietary fat, carbohydrate, dietary fiber, and dietary potassium; ^b^ Model 2: based on Model 1, adjusted for total energy, protein, dietary fat, carbohydrate, dietary fiber, and dietary sodium; ^c^ Model 2: based on Model 1, adjusted for total energy, protein, dietary fat, carbohydrate, and dietary fiber; Model 3: based on Model 2, adjusted for BMI. * *p* < 0.05, ** *p* < 0.01.

## Supplementary Materials

The following are available online at https://www.mdpi.com/2072-6643/12/2/366/s1, Table S1: Baseline characteristics of participants according to area quartiles of dietary potassium intake.
