# Supplementary material for: Associations of Dietary Sodium, Potassium, and Sodium to Potassium Ratio with Blood Pressure—Regional Disparities in China
Source: Nutrients. 2020 Jan 30;12(2):366. doi: 10.3390/nu12020366 (PMC7071283; doi:10.3390/nu12020366)
Supplement: Supplementary file 1 [file nutrients-12-00366-s001.pdf]

Table S1 Baseline characteristics of participants according to areas quartiles of dietary potassium intake.

**Characteristics**	**North China**				p	**South China**				p
	**Q1**	**Q2**	**Q3**	**Q4**		**Q1**	**Q2**	**Q3**	**Q4**	
N	652	653	653	652		1024	1024	1024	1023	
Potassium(mg/d)	1093.5 (944.2, 1198)	1450.5 (1370.6, 1532.2)	1824.7 (1721.9, 1937.2)	2384.6 (2178, 2797.3)	< 0.0001	977.2 (826.1, 1077.4)	1346.2 (1265.8, 1436)	1706.4 (1606.8, 1830.8)	2346.1 (2118.6, 2786.8)	< 0.0001
Age(years)[a]	47.6 ± 14.3	45.8 ± 13	45.8 ± 12.6	46.1 ± 12.6	0.1231	50.1 ± 15.1	47.4 ± 14.0	47.1 ± 13.9	47.0 ± 14.0	< 0.0001
Household income, n (%)					< 0.0001					< 0.0001
Low	252 (38.83)	219 (33.74)	207 (31.8)	188 (29.06)		418 (41.59)	355 (34.94)	314 (30.91)	264 (25.93)	
Medium	201 (30.97)	242 (37.29)	213 (32.72)	209 (32.3)		331 (32.94)	311 (30.61)	330 (32.48)	381 (37.43)	
High	196 (30.2)	188 (28.97)	231 (35.48)	250 (38.64)		256 (25.47)	350 (34.45)	372 (36.61)	373 (36.64)	
Urbanicity index, n (%)					< 0.0001					< 0.0001
Low	177 (27.11)	224 (34.36)	248 (37.98)	216 (33.13)		333 (32.55)	330 (32.23)	308 (30.08)	377 (36.82)	
Medium	271 (41.5)	209 (32.06)	185 (28.33)	202 (30.98)		402 (39.3)	363 (35.45)	342 (33.4)	274 (26.76)	
High	205 (31.39)	219 (33.59)	220 (33.69)	234 (35.89)		288 (28.15)	331 (32.32)	374 (36.52)	373 (36.43)	
Physical activity, n (%)					0.0005					0.0961
Low	265 (40.58)	217 (33.28)	195 (29.91)	191 (29.29)		373 (36.46)	342 (33.4)	342 (33.43)	307 (29.98)	
Medium	196 (30.02)	216 (33.13)	224 (34.36)	235 (36.04)		327 (31.96)	337 (32.91)	350 (34.21)	350 (34.18)	
High	192 (29.4)	219 (33.59)	233 (35.74)	226 (34.66)		323 (31.57)	345 (33.69)	331 (32.36)	367 (35.84)	
Smoking, n (%)					< 0.0001					0.0043
No	516 (79.02)	480 (73.62)	442 (67.69)	422 (64.72)		739 (72.24)	738 (72.07)	683 (66.7)	691 (67.48)	
Yes	136 (20.83)	171 (26.23)	211 (32.31)	230 (35.28)		284 (27.76)	284 (27.73)	341 (33.3)	333 (32.52)	
Drinking, n (%)					< 0.0001					< 0.0001
No	494 (75.65)	445 (68.25)	417 (63.86)	375 (57.52)		766 (74.88)	727 (71)	636 (62.11)	610 (59.57)	
Yes	159 (24.35)	207 (31.75)	236 (36.14)	277 (42.48)		257(25.12)	295 (28.81)	388 (37.89)	414 (40.43)	
BMI (kg/m^2) [b]	23.3 (21.4, 25.7)	23.2 (21.1, 25.5)	23.6 (21.3,26)	23.4 (21.4, 25.6)	0.1654	22.2 (20.1, 24.3)	22.2 (20.5, 24.5)	22.6 (20.4, 24.9)	22.4 (20.6, 24.6)	0.0156
SBP (mmHg) [b]	119 (110, 123)	120 (110, 123)	120 (110, 124)	119 (110, 122.5)	0.0625	118 (110, 125)	117 (109, 122)	116 (108, 123)	118 (109, 124)	0.005
DBP (mmHg) [b]	80 (72, 81)	79 (71, 81)	79 (72, 81)	80 (71, 81)	0.9082	75 (70, 80)	76 (70, 80)	75 (70, 80)	76 (70, 80)	0.0599
Total energy(kcal/d) [b]	1536.0 (1291.9, 1798.4)	1893.5 (1605.6, 2127.3)	2208.7 (1864.6, 2523.1)	2580.5 (2207.2, 3000.5)	< 0.0001	1557.4 (1245.3, 1898.4)	1944.8 (1620, 2271.7)	2194.6 (1840.7, 2589)	2659.3 (2228.9, 3172.7)	< 0.0001
Protein(g/d) [b]	45.1 (38.1, 53.5)	57.2 (49.7, 65.1)	68.5 (57.8, 78.7)	85.1 (71.8, 102.8)	< 0.0001	43.6 (35.6, 51.5)	57.4 (50.4, 67.1)	70.3 (61, 80.6)	89.0 (75.7, 106.8)	< 0.0001
Dietary fat(g/d) [b]	53.2 (38.2, 72.2)	62.3 (45.6, 82.0)	72.9 (54.7, 95.1)	87.8 (64.2, 114.4)	< 0.0001	59.9 (42.4, 78.3)	73.1 (54.4, 96.7)	84.4 (63.3, 108.3)	99.9 (76.7, 126.0)	< 0.0001
Carbohydrate(g/d) [b]	203.3 (163.2, 249.9)	254.5 (204.6, 308.1)	293.2 (237.8, 363)	338.2 (266.9, 424.2)	< 0.0001	196 (140.4, 247.8)	243.4 (180.3, 299.6)	264.1 (194.7, 338.2)	321.9 (249.8, 415.3)	< 0.0001
Dietary fiber(g/d) [b]	7.8 (6.3, 9.7)	10.0 (8.6, 12.2)	12.5 (10.4, 15.2)	16.4 (12.9, 21)	< 0.0001	5.7 (4.4, 7.3)	7.9 (6.6, 9.9)	10.0 (8.2, 12.7)	15.7 (11.9, 21.5)	< 0.0001
Sodium (mg/d) [b]	3817.3 (2694.8, 5292.9)	4178.8 (3048.3, 5617.9)	4511.1 (3167.8, 6333.8)	5167.6 (3642.6, 6993.3)	< 0.0001	3385.1 (2289.4, 4802.7)	3911.4 (2900.9, 5338.3)	4322.2 (3174.3, 6014.7)	4908.9 (3572, 6914.2)	< 0.0001

Abbreviation: Q = quartile, SBP = systolic blood pressure, DBP = diastolic blood pressure. Data of categorical variables expressed as number (%); [a] Mean ± SE. [b] Median (interquartile ranges) for skewed distribution variable.
